# Association of Glucose-Lowering Drugs With Outcomes in Patients With Diabetes Before Hospitalization for COVID-19: A Systematic Review and Network Meta-analysis

**DOI:** 10.1001/jamanetworkopen.2022.44652

**Published:** 2022-12-06

**Authors:** Zheng Zhu, Qingya Zeng, Qinyu Liu, Junping Wen, Gang Chen

**Affiliations:** 1 Shengli Clinical Medical College of Fujian Medical University, Fuzhou, Fujia, China; 2 Department of Endocrinology, Fujian Provincial Hospital, Fuzhou, Fujian, China; 3 Fujian Provincial Key Laboratory of Medical Analysis, Fujian Academy of Medical Sciences, Fuzhou, Fujian, China

## Abstract

**Importance:**

Patients with COVID-19 have a high prevalence of diabetes, and diabetes and blood glucose control are determinants of intensive care unit admission and mortality.

**Objective:**

To evaluate the association between COVID-19–related adverse outcomes and 8 antihyperglycemic drugs in patients with diabetes who were subsequently diagnosed and hospitalized with COVID-19.

**Data Sources:**

Data were retrieved and collected in PubMed, Embase, Cochrane Central Register, Web of Science, and ClinicalTrials.gov from database inception to September 5, 2022.

**Study Selection:**

For this systematic review and network meta-analysis, randomized clinical trials and observational studies conducted among patients with diabetes while receiving glucose-lowering therapies for at least 14 days before the confirmation of COVID-19 infection were included after blinded review by 2 independent reviewers and consultations of disagreement by a third independent reviewer. Of 1802 studies initially identified, 31 observational studies met the criteria for further analysis.

**Data Extraction and Synthesis:**

This study follows the Preferred Reporting Items for Systematic Reviews and Meta-analyses reporting guideline. Bayesian network meta-analyses were performed with random effects.

**Main Outcomes and Measures:**

A composite adverse outcome, including the need for intensive care unit admission, invasive and noninvasive mechanical ventilation, or in-hospital death.

**Results:**

Thirty-one distinct observational studies (3 689 010 patients with diabetes hospitalized for COVID-19) were included. The sodium-glucose cotransporter-2 inhibitors (SGLT-2is) were associated with relatively lower risks of adverse outcomes compared with insulin (log of odds ratio [logOR], 0.91; 95% credible interval [CrI], 0.57-1.26), dipeptidyl peptidase-4 inhibitors (logOR, 0.61; 95% CrI, 0.28-0.93), secretagogues (logOR, 0.37; 95% CrI, 0.02-0.72), and glucosidase inhibitors (logOR, 0.50; 95% CrI, 0.00-1.01). Based on the surface under the cumulative ranking curves value, SGLT-2is were associated with the lowest probability for adverse outcomes (6%), followed by glucagon-like peptide-1 receptor agonists (25%) and metformin (28%). A sensitivity analysis revealed that the study was reliable.

**Conclusions and Relevance:**

These findings suggest that the use of an SGLT-2i before COVID-19 infection is associated with lower COVID-19–related adverse outcomes. In addition to SGLT-2is, glucagon-like peptide-1 receptor agonists and metformin were also associated with relatively low risk of adverse outcomes.

## Key Points

**Question:**

What is the difference in the association between COVID-19–related adverse outcomes and 8 routine glucose-lowering therapies in hospitalized patients with diabetes?

**Findings:**

In this network meta-analysis of 31 observational studies with more than 3.6 million patients, sodium-glucose cotransporter-2 inhibitors were associated with lower risk of COVID-19–related adverse outcomes in diabetes, followed by glucagon-like peptide-1 receptor agonists and metformin, compared with insulin, dipeptidyl peptidase-4 inhibitors, secretagogues, and glucosidase inhibitors.

**Meaning:**

The findings of this meta-analysis provide information regarding routine antihyperglycemic medications and COVID-19–related adverse outcomes.

## Teaser

This meta-analysis reviews the associations between different glucose-lowering therapies and outcomes among patients with diabetes who received therapy before being hospitalized with COVID-19.

## Introduction

Patients with COVID-19 have a high prevalence of diabetes, and diabetes and blood glucose control are determinants of intensive care unit admission and mortality.^1,2^ The risk and severity of infection in patients with diabetes and COVID-19 are associated with increased angiotensin-converting enzyme 2 expression, increased furin levels, impaired T-cell function, and increased interleukin (IL)-6,^1^ which makes it possible for diabetes to promote COVID-19 infection because of increased viral entry into cells and impaired immune response.^1^

So far, sodium-glucose cotransporter-2 inhibitors (SGLT-2is), glucagon-like peptide-1 receptor agonists (GLP-1RAs), metformin, dipeptidyl peptidase-4 inhibitors (DPP-4is), thiazolidinediones, secretagogues, α-glucosidase inhibitors (AGIs), and insulin have been used as routine glucose-lowering therapies. Studies on COVID-19 have found that metformin is not as harmful as expected and perhaps has possible benefits.^3^ A previous human study found that pioglitazone can significantly reduce lung inflammation markers, including IL-6, IL-8, and tumor necrosis factor α.^4^ Another study found that the mortality of patients with diabetes and COVID-19 receiving a DPP-4i was low.^5^ In addition, SGLT-2is may favorably affect the dysregulated process in the setting of a COVID-19 cytokine storm, but some experts recommended against the use of an SGLT-2i in patients with COVID-19 because of the risk of dehydration and euglycemic diabetic ketoacidosis.^6^ Experimental studies also showed that liraglutide had anti-inflammatory effects on acute lung injury,^7^ but liraglutide has the theoretical concern of increasing the expression of angiotensin-converting enzyme 2 combining with COVID-19.^8^ A large number of meta-analyses have recently shown that metformin, GLP-1RAs, SGLT-2is, secretagogues, and DPP-4is were associated with lower adverse outcomes in patients with diabetes and COVID-19.^3,9,10,11^ However, there is a lack of network meta-analyses of studies that compare SGLT-2is, GLP-1RAs, metformin, DPP-4is, thiazolidinediones, secretagogues, AGIs, and insulin. To select potential antidiabetes medications that could improve outcomes, we conducted a network meta-analysis to evaluate the association of COVID-19–related adverse outcomes and 8 glucose-lowering therapies for patients with diabetes before the confirmation of COVID-19 infection.

## Methods

We conducted a systematic review and network meta-analysis following the Preferred Reporting Items for Systematic Reviews and Meta-analyses (PRISMA) reporting guideline (eAppendix 1 in the Supplement). Details of data information collection are shown in eAppendix 2 in the Supplement.

### Search Strategy and Study Selection

The following electronic databases were searched from database inception to September 5, 2022: PubMed, Embase, Cochrane Central Register, Web of Science, and ClinicalTrials.gov. In addition, hand-searching was performed to include any relevant studies that were not shown in the initial database search. The reference lists of included original research, literature reviews, and meta-analyses were also screened to identify any other potential studies that could be used, and we obtained raw data by contacting the corresponding author. The detailed search strategy used in PubMed is available in eTable 1 in the Supplement.

Observational studies (cohort or case-control design) and randomized clinical trials (RCTs) were included in this meta-analysis. If the number of RCTs was insufficient to form a network, systematic reviews were used to describe them. Studies had to include at least 2 of the following glucose-lowering therapies as interventions: SGLT-2is, GLP-1RAs, metformin, DPP-4is, thiazolidinediones, secretagogues, AGIs, and insulin. Study participants were patients with diabetes hospitalized for COVID-19 who received glucose-lowering therapies for 14 days before hospitalization. Studies that had a composite adverse outcome of the need for intensive care unit admission, invasive and noninvasive mechanical ventilation, or in-hospital death were included in this meta-analysis. If all 3 outcomes were described, we examined in-hospital death data. Exclusion criteria were incomplete data; reviews, comments, editorials, and letters to the editor; and non–peer-reviewed studies. If the reported data overlapped, articles with the most complete data were included. The study searching and selection, data extraction, and risk of bias assessment were conducted independently by 2 reviewers (Z.Z. and Q.Y.Z.). Any differences were resolved through discussion or consultation with a third independent reviewer.

### Data Extraction and Bias Risk Assessment

We extracted the following information from each study: study information, participant characteristics at baseline, and total number and number of deaths of patients using each glucose-lowering therapy. The Newcastle-Ottawa Scale^12^ was used to assess the risk of bias in nonrandomized studies. The risk of bias was divided into 3 main aspects: selection, comparability, and exposure. The highest score for each study was 9; studies with a score of 7 or higher were considered to be of high quality, and articles of poor quality (score >0 to <4) were excluded.

### Statistical Analysis

#### Summary Treatments and Synthesis of Results

Stata software, version 15.1 (StataCorp LLC) was used to draw a network diagram in which each node represented a certain intervention, node size represented the sample size, and the line thickness represented the number of studies that compared every pair of treatments. Using a bayesian approach, we performed a random-effects network meta-analysis (R software, version 4.1.0 [R Foundation for Statistical Computing] with the gemtc package) to compare adverse outcomes for medications.^13^ Pairwise comparisons from each model were made using relative effect tables with adverse outcome expressed as log of odds ratio (logOR), in which negative values indicate superiority.

#### Model Fitting and Consistency Evaluation

We checked the model fit by comparing the sum of the leverages of each data point, the deviance information criterion according to the GeMTC manual,^14^ and the random-effects standard of consistency and inconsistency models to reconfirm that we chose the correct model. To evaluate the consistency of evidence, a node-splitting approach was also performed for each comparison in the treatment network with actual trial data (treatment effects estimated by direct evidence) and inferred data (treatment effects estimated using indirect evidence).^15,16^ In this approach, 1 treatment comparison is split into parameters of direct and indirect evidence to assess whether they are consistent (between-trial differences in the underlying treatment effects).^17^

#### Convergence Evaluation

A potential scale reduced factor (PSRF) derived from the Brooks-Gelman-Rubin Diagnosis Plot reflected the convergence of the model, with a PSRF of 0% and 100% indicating the worst and best treatments, respectively.^18,19^ Applying generalized linear models with a log-link function, we used the Markov Chain Monte Carlo method with 50 000 burn-in and an additional 100 000 simulations with 4 chains of different initial values to obtain medians and 95% credible intervals (CrIs).^20^

#### Rank Probabilities

To assess the likelihood that a given glucose-lowering therapy is the best, second best, and so on within a network, rank probabilities were determined and converted to cumulative rank probabilities from which surface under the cumulative ranking (SUCRA) curves were generated. The smaller the SUCRA value (ranging from 0 to 1), the lower the chance of a respective adverse event.^21,22^ In addition, we generated figure-ranking probabilities for all antihyperglycemics.

#### Publication Bias, Heterogeneity Evaluation, and Sensitivity Analysis

A funnel plot was used to evaluate publication bias. Heterogeneity was assessed in dichotomous direct comparisons with Cochran *Q* and *I*^2^ when 2 or more direct comparisons between classes were available. Finally, we checked the sensitivity of our model by rerunning our adverse outcome analysis without the treatment with the highest-scoring SUCRA value and then again without the lowest-scoring SUCRA value. Metaregression analysis was performed to account for the potential effect modifiers for the mean age and sex on the pooled outcomes.

## Results

### Search Results and Study Characteristics

**Figure 1.**
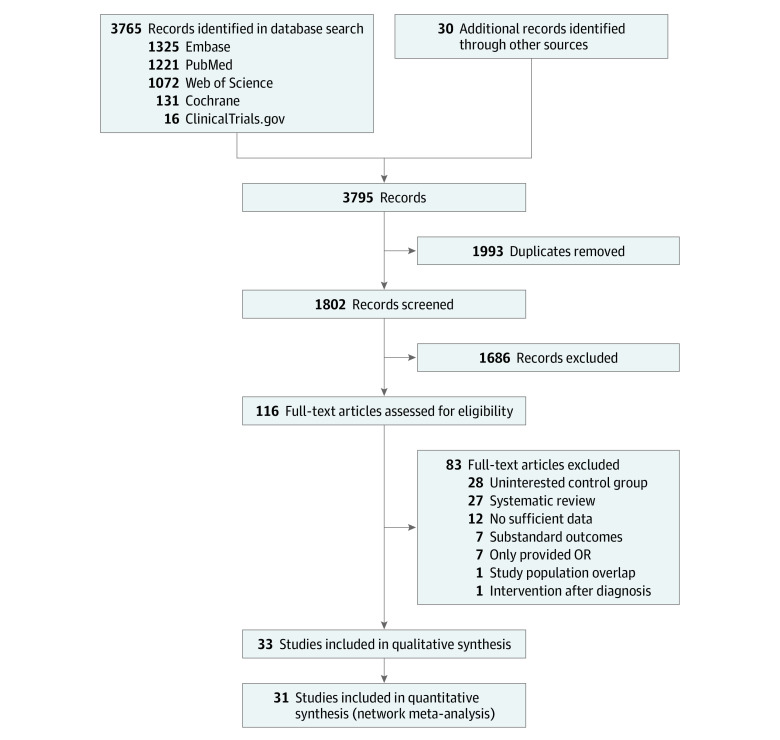
Flowchart According to the Preferred Reporting Items for Systematic Reviews and Meta-analyses (PRISMA) Guideline for the Systematic Literature Search OR indicates odds ratio.

**Figure 2.**
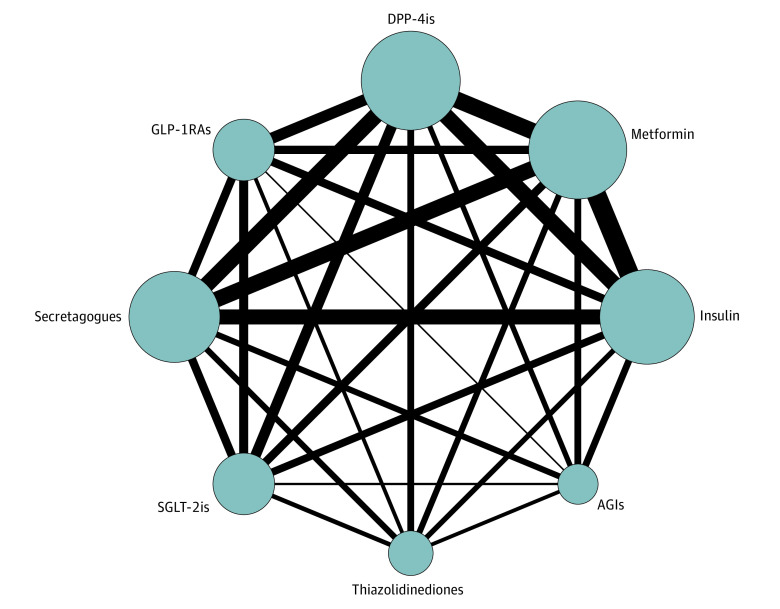
Structure of the Network Formed by Interventions and Both Direct and Indirect Comparisons for the Outcomes This network diagram shows comparisons of antihyperglycemics in observational studies with respect to the sample sizes and number of studies. Each node represents a certain intervention, node size represents the sample size, and line thickness represents the number of studies comparing every pair of treatments: insulin (26 studies, 356 235 patients), metformin (27 studies, 1 809 622 patients), dipeptidyl peptidase-4 inhibitors (DPP-4is; 26 studies, 488 072 patients), glucagon-like peptide-1 receptor agonists (GLP-1RAs; 15 studies, 110 223 patients), secretagogues (22 studies, 568 583 patients), sodium-glucose cotransporter-2 inhibitors (SGLT-2is; 14 studies, 271 389 patients), thiazolidinediones (9 studies, 60 651 patients), and α-glucosidase inhibitors (AGIs; 9 studies, 2229 patients).

The flowchart of the literature selection is shown in Figure 1. Of 1802 studies initially identified, 31 distinct observational studies^23,24,25,26,27,28,29,30,31,32,33,34,35,36,37,38,39,40,41,42,43,44,45,46,47,48,49,50,51,52,53^ (3 689 010 patients with diabetes hospitalized for COVID-19) were included in this network meta-analysis based on the selection criteria. The evidence network comprising 8 glucose-lowering therapies is shown in Figure 2. Among these were 148 active treatment groups with 26 insulin groups, 27 metformin groups, 26 DPP-4i groups, 15 GLP-1RA groups, 22 secretagogue groups, 14 SGLT-2i groups, 9 thiazolidinedione groups, and 9 AGI groups. Five studies^25,30,34,37,43^ involved more than 7 treatment groups. Eighteen studies had 4 to 6 groups,^23,24,27,28,33,35,36,39,40,41,44,45,46,47,49,50,52,53^ and 5 studies had 3 groups.^26,31,38,42,48^ The characteristics of the included studies are listed in the Table. The mean age was similar across the studies (range, 55-85 years). The proportion of male participants was between 32% and 72% except for the study by Lally et al,^29^ which reported almost all male patients from communities of the Veterans Health Administration. For most studies, diabetes duration was more than 10 years. Body mass index (calculated as weight in kilograms divided by height in meters squared) ranged from 23 to 30 or greater, and the mean hemoglobin A_1c_ baseline levels ranged from 7.1% to greater than 8.7% (to convert to proportion of total hemoglobin, multiply by 0.01). A total of 22 006 adverse outcomes occurred. Most of these individuals used metformin before the confirmation of COVID-19 (37.09%); nearly one-fifth used insulin (19.53%) and DPP-4is (19.54%), followed by secretagogues (14.74%), GLP-1RAs (3.94%), SGLT-2is (3.33%), thiazolidinediones (1.52%), and AGIs (0.33%).

**Table.** Characteristics of Studies Included in the Network Meta-analysis

Source	Age, y	Male, %	BMI	HbA_1c_, %	Diabetes duration, y	Glucose-lowering therapies, No. of users/No. of outcomes								NOS score
						Insulin	Metformin	DPP-4is	GLP-1RAs	Secretagogues	SGLT-2is	Thiazolidinediones	AGIs	
Yan et al,^23^ 2020	56	50.7	24.01	NR	NR	15/8	26/8	6/1	NR	15/5	NR	3/2	16/7	8
Wargny et al,^24^ 2021	69.7	63.7	28.4	7.7	11	1039/285	1553/434	615/169	254/76	782/235	NR	NR	NR	8
Silverii et al,^25^ 2021	NR	NR	NR	NR	NR	43/18	76/21	13/5	7/1	33/13	4/2	8/4	NR	9
Pérez-Belmonte et al,^26^ 2020	74.9	61.9	29.0^a^	NR	NR	129/57	249/91	105/45	NR	NR	NR	NR	NR	9
Mirani et al,^27^ 2020	71	72.2	47.8^a^	NR	NR	29/19	69/25	11/1	NR	NR	NR	NR	NR	7
Li et al,^28^ 2020	66.8	56.5	24.23	7.89	NR	26/3	37/2	NR	NR	22/3	NR	NR	38/3	9
Lally et al,^29^ 2021	72.4	97.8	29.5	7.58	NR	103/24	127/16	NR	NR	NR	NR	NR	NR	9
Khunti et al,^30^ 2021	65	58.3	51.4^a^	46.9^b^	59.2^c^	350 960/ 2825	1 800 005/ 6295	479 555/ 2733	100 820/ 279	565 730/ 2648	266 505/ 458	60 085/ 226	1665/15	9
Israelsen et al,^31^ 2021	60	58.4	NR	NR	10	NR	NR	284/30	370/14	NR	520/16	NR	NR	7
Crouse et al,^32^ 2021	NR	NR	NR	NR	NR	87/15	76/8	NR	NR	NR	NR	NR	NR	8
Chen et al,^33^ 2020	66	NR	NR	8.1	NR	27/8	22/4	20/5	NR	53/7	NR	NR	69/11	8
Zhang et al,^34^ 2020	62	60	23.6	NR	NR	37/12	20/1	2/0	NR	12/2	2/0	4/0	31/6	7
Cariou et al,^35^ 2020	69.8	64.9	28.4	8.1	NR	504/70	746/63	285/27	123/9	367/32	NR	NR	NR	8
Elibol et al,^36^ 2021	63.3	45.6	NR	NR	6.2	NR	379/74	246/56	NR	66/14	56/14	27/7	NR	8
Hayek et al,^37^ 2021	65.3	49.8	30.7	7.24	55.5^c^	1328/317	3770/598	193/59	667/100	384/81	403/76	174/26	NR	8
Kahkoska et al,^38^ 2021	58.4	46.9	35.5	8	NR	NR	NR	3511/217	6692/153	NR	3665/91	NR	NR	8
Luk et al,^39^ 2021	65.9	56.3	24	7.5	2	385/32	737/44	199/18	NR	385/35	NR	NR	NR	8
Luo et al,^40^ 2021	64	56.5	NR	7.87	NR	88/13	54/3	11/1	NR	37/1	NR	7/0	77/2	9
Sourij et al,^41^ 2021	71.1	63.9	29.1	NR	NR	52/12	77/14	42/15	3/0	14/6	24/3	NR	NR	8
Nyland et al,^42^ 2021	61.5	46.4	33.3	8.11	NR	NR	NR	2264/667	1163/183	NR	NR	340/68	NR	7
Ong et al,^43^ 2021	62.7	55.8	27.7	7.3	NR	52/16	143/30	121/32	3/0	50/9	26/3	3/1	1/0	7
Orioli et al,^44^ 2021	69	48	30.5	7.1	NR	31/5	45/4	4/0	5/0	19/1	4/0	NR	NR	8
Pazoki et al,^45^ 2021	65.4	56.2	28	NR	NR	53/16	177/48	20/5	NR	72/23	NR	NR	NR	8
Ramos-Rincón et al,^46^ 2021	85.9	47.1	17.7^a^	NR	NR	211/103	420/206	253/110	24/11	NR	32/17	NR	NR	8
Shestakova et al,^47^ 2020	64.4	32.4	32.3	7.4	9.6	114/27	196/17	26/4	1/0	129/15	13/0	NR	NR	7
Zhang et al,^48^ 2020	62	48.6	24.54	8.7	NR	43/19	25/4	NR	NR	NR	NR	NR	37/14	8
Yu et al,^49^ 2021	65.2	54.1	NR	7.7	NR	346/94	223/8	50/1	NR	109/0	NR	NR	295/14	8
Nafakhi et al,^50^ 2021	60	43	29.8	NR	NR	15/4	35/2	14/2	NR	24/2	NR	NR	NR	8
Cheng et al,^51^ 2021	56	54	25.56	NR	NR	2/11	18/1	NR	NR	NR	NR	NR	NR	7
Yeh et al,^52^ 2022	65.8	47.6	32.4	7.6	NR	507/293	317/140	92/64	29/25	149/79	82/41	NR	NR	8
Min et al,^53^ 2022	NR	NR	NR	NR	NR	NR	NR	130/32	62/17	121/29	53/11	NR	NR	8

Abbreviations: AGIs, α-glucosidase inhibitors; BMI, body mass index (calculated as weight in kilograms divided by height in meters squared); DPP-4is, dipeptidyl peptidase-4 inhibitors; GLP-1RAs, glucagon-like peptide-1 receptor agonists; HbA_1c_, hemoglobin A_1c_ (to convert to proportion of total hemoglobin, multiply by 0.01); NOS, Newcastle-Ottawa Scale; NR, not reported; SGLT-2is, sodium-glucose cotransporter-2 inhibitors.

^a^ The percentage of patients with BMI of 30 or greater.

^b^ The percentage of patients with HbA_1c_ of 7.5% or greater.

^c^ The percentage of patients with diabetes duration of 10 years or more.

In addition, 2 RCTs^54,55^ had completed research and published the results. Abuhasira et al^54^ conducted a multicenter RCT of linagliptin and standardized treatment on 64 inpatients with diabetes and COVID-19 in 3 Israeli hospitals. The study found no difference in the time of clinical improvement compared with the standard of care. In addition, a parallel, double-blind RCT^55^ examined treatment with linagliptin and insulin or insulin alone in 73 inpatients with COVID-19 and hyperglycemia. In this study, Guardado-Mendoza et al^55^ found that the combination of linagliptin and insulin reduced the relative risk of assisted mechanical ventilation by 74% but found no difference in the risk of death after a follow-up of 30 days. Based on the results of 2 RCTs,^54,55^ the effect of DPP-4is on adverse outcome of diabetes with COVID-19 is still unclear. Given the limited number of RCTs, we conducted a network meta-analysis of observational studies to obtain evidence from studies with a large community-based sample size to generalize the findings to the general population.

### Quality Assessment of the Included Studies

Seven case-control studies^27,36,40,43,47,48,50^ and 24 cohort studies^23,24,25,26,28,29,30,31,32,33,34,35,37,38,39,41,42,44,45,46,49,51,52,53^ were included. All 31 included studies had Newcastle-Ottawa Scale scores higher than 7, indicating no risk of bias in our analysis. The detailed results of the risk of bias assessment are given in eTable 2 in the Supplement.

### Major Results of the Network Meta-analysis

**Figure 3.**
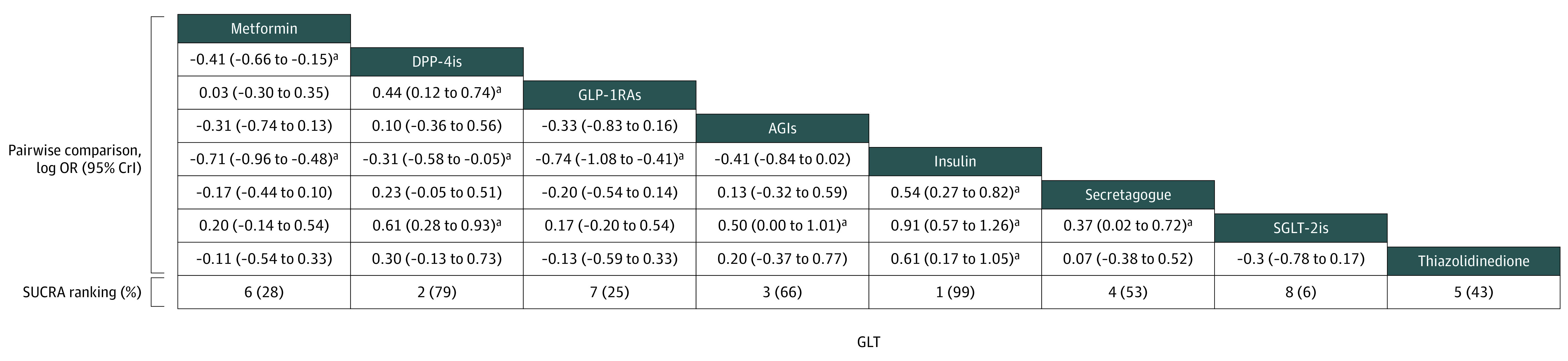
Pairwise Comparisons of Glucose-Lowering Therapies Adverse outcomes reported as log of odds ratio (95% credible interval), with negative values indicating superiority of the noted therapy. Rankings according to the surface under the cumulative ranking curve (SUCRA) hierarchy appear in the last row, with 1 denoting the most likely risk of adverse outcomes associated with treatment and SUCRA percentage appearing in parentheses. AGIs indicates α-glucosidase inhibitors; DPP-4is, dipeptidyl peptidase-4 inhibitors; GLP-1RAs, glucagon-like peptide-1 receptor agonists; and SGLT-2is, sodium-glucose cotransporter-2 inhibitors. ^a^Significant difference.

Pairwise comparisons revealed significant differences between glucose-lowering therapies (Figure 3; eFigure 1 in the Supplement). The SGLT-2is were superior to the DPP-4is (logOR, 0.61; 95% CrI, 0.28-0.93), AGIs (logOR, 0.50; 95% CrI, 0.00-1.01), insulin (logOR, 0.91; 95% CrI, 0.57-1.26), and secretagogues (logOR, 0.37; 95% CrI, 0.02-0.72). The GLP-1RAs were superior to the DPP-4is (logOR, 0.44; 95% CrI, 0.12-0.74) and insulin (logOR, 0.74; 95% CrI, 0.41-1.08). Insulin was inferior to metformin (logOR, 0.71; 95% CrI, 0.48-0.96), DPP-4is (logOR, 0.31; 95% CrI, 0.05-0.58), secretagogues (logOR, 0.54; 95% CrI, 0.27-0.82), and thiazolidinediones (logOR, 0.61; 95% CrI, 0.17-1.05). The SGLT-2is were associated with a lower risk of adverse outcomes, followed by the GLP-1RAs and metformin, whereas insulin was associated with a higher risk of adverse outcomes.

### SUCRA Scores

**Figure 4.**
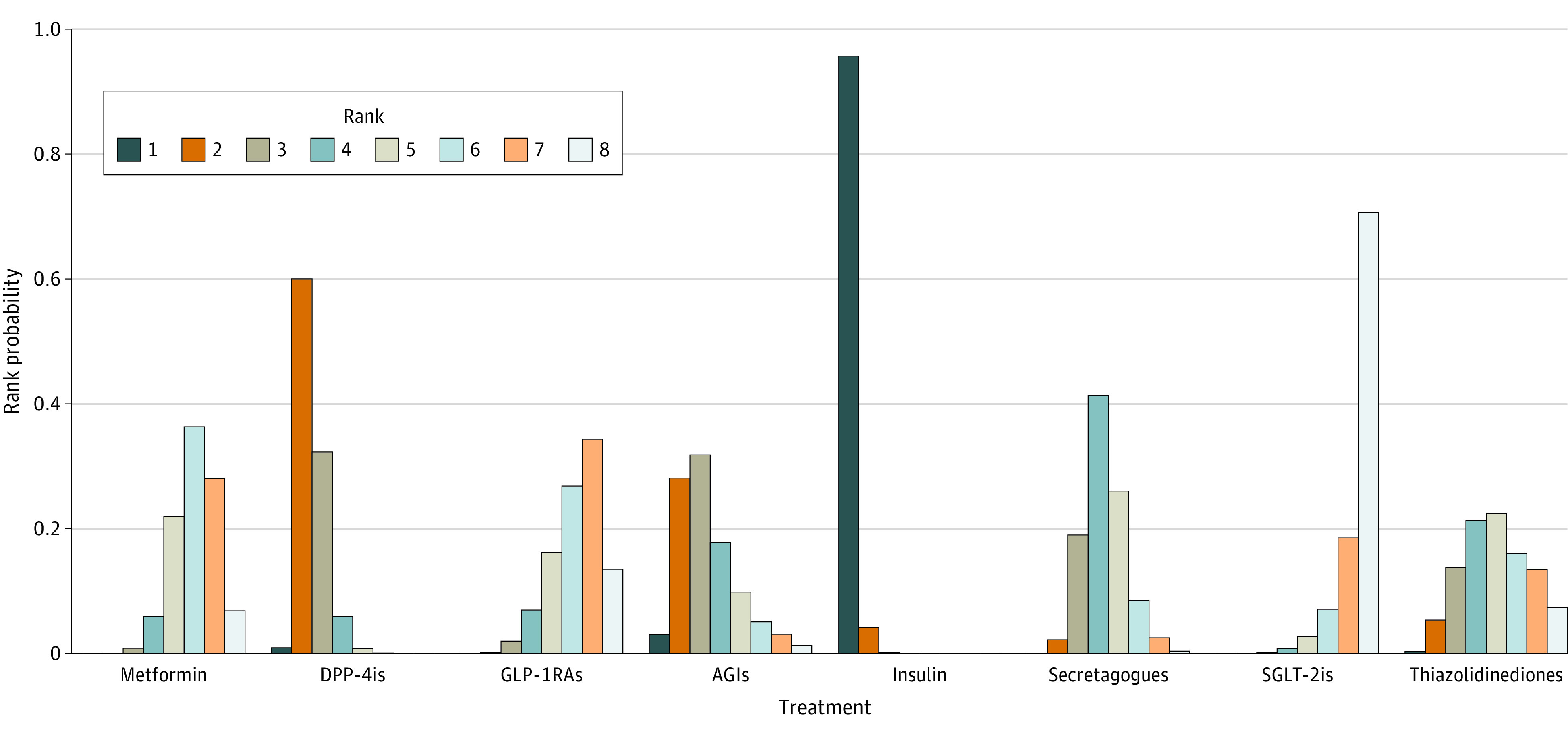
Rank Probability of Strategy Interventions All 8 therapies were ranked based on their probabilities of increasing adverse outcomes. The higher the rank, the lower risk of the outcomes. For example, rank 8 is better than rank 1. AGIs indicates α-glucosidase inhibitors; DPP-4is, dipeptidyl peptidase-4 inhibitors; GLP-1RAs, glucagon-like peptide-1 receptor agonists; and SGLT-2is, sodium-glucose cotransporter-2 inhibitors.

The SUCRA scores for adverse outcomes showed a ranking consistent with that of the ORs (Figure 3). Combined with Figure 4, for the possibility of adverse outcomes, SGLT-2is had the highest probability of ranking eighth, GLP-1RAs ranked seventh, and metformin ranked sixth, whereas insulin had the highest probability of ranking first. According to rank probability, SGLT-2is, GLP-1RAs, and metformin were associated with lower risk of adverse outcomes compared with insulin.

### Consistency and Heterogeneity Tests

The difference of deviance information criterion value between the consistent model and inconsistent model was 1.342 (difference of deviance information criterion <5) (eTable 3 in the Supplement). The node-split modeling analysis of the outcome showed that there was good local consistency between direct and indirect comparisons (eTable 4 in the Supplement). The funnel plots of these studies appear to be symmetric, indicating no publication bias (eFigure 2 in the Supplement). As for heterogeneity, the overall *I*^2^ of each analysis was no more than 47% (*I*^2^ < 50%), which can be ignored (eTable 5 in the Supplement).

### Convergence Evaluation

The convergence evaluation results showed that the PSRFs were all equal to 1 (eFigure 3 in the Supplement). According to the plots and the PSRF, simulation times had reached a good convergence, and statistical results were reliable.

### Sensitivity Analysis

The sensitivity analysis revealed that our study was reliable. When the medication with the lowest SUCRA value (SGLT-2is) was removed, there was no change in the relative ranking of the remaining medications. When the medication with the highest SUCRA value (insulin) was removed, AGIs shifted in ranking from third to second and DPP-4is from second to third (eTable 6 in the Supplement). In addition, network metaregression analysis revealed that the association between adverse outcomes and 8 glucose-lowering therapies did not differ by mean age and sex (eFigure 4 in the Supplement).

## Discussion

This network meta-analysis was based on 31 studies that involved 3 689 010 individuals and compared the association between the risk of COVID-19–related adverse outcomes and 8 glucose-lowering therapies in patients with diabetes before diagnosis of COVID-19. The principal findings of our study were that, compared with insulin, DPP-4is, secretagogues, glucosidase inhibitors, thiazolidinediones, and SGLT-2is were associated with lower COVID-19–related adverse outcomes in patients with diabetes and that, in addition to SGLT-2is, GLP-1RAs and metformin were also associated with relatively low risk of adverse outcomes.

It is unclear whether SGLT-2is should be used as glucose-lowering therapy during the COVID-19 pandemic because of the risk of dehydration and euglycemic diabetic ketoacidosis.^56^ Based on the updated evidence, however, Khunti et al^57^ and Koufakis et al^58^ proposed to reexamine the widespread policy of stopping use of SGLT-2is during acute illness. Thus, accumulating evidence suggests that the benefits of SGLT-2is go beyond the improvement of glycemic control and have potential cardiovascular and kidney advantages.^59,60,61^ For instance, EMPEROR-Reduced (Empagliflozin Outcome Trial in Patients With Chronic Heart Failure With Reduced Ejection Fraction) showed that empagliflozin markedly reduced the end points of cardiovascular death and heart failure and was associated with lower serious kidney outcomes.^62^ In addition to increasing fatty acid oxidation, SGLT-2is can improve mitochondrial function and insulin sensitivity, enhance the organ’s ability to resist physiologic stress, and have potential therapeutic activities on hypertension and obesity.^63^ In addition, SGLT-2is reduced mortality in experimental pulmonary hypertension, in part because of the observed reduced pulmonary remodeling.^64^ We know that cardiometabolic comorbidities and their underlying obesity and insulin resistance may increase further impairment of oxidative stress, inflammation, and metabolic disorders in patients with COVID-19.^65^ Dapagliflozin can reduce the risk of cardiovascular events.^66^ Thus, SGLT-2is may be related to ameliorating COVID-19 risk factors in this context. Our results suggest that compared with other diabetes drugs, the use of SGLT-2is before COVID-19 infection in patients with diabetes was associated with a lower incidence of adverse outcomes after infection, which may be associated with improving blood glucose level, blood pressure, body weight, and lipid metabolism. However, the number of participants using SGLT-2is in our study was relatively small, so more participants and RCTs are needed to further verify this view.

Our study also found that GLP-1RAs and metformin were associated with a relatively low risk of adverse outcomes. Some researchers have speculated that GLP-1RAs were a candidate for treatment of patients with or without diabetes with COVID-19 owing to their multiple beneficial effects on excessive inflammation-induced acute lung injury.^67^ Excessive inflammatory responses, such as cytokine storms and disseminated thromboembolic events, are considered fatal complications of COVID-19 infection.^68^ In addition to the expected improvement in blood glucose control or obesity, metformin has also been shown to have antifibrinolytic activities and inhibit inflammatory cytokines.^69,70^ Therefore, some researchers speculated that metformin might play a role in the immune response to COVID-19, which might improve the prognosis.^71^

The result that needs further evaluation is that insulin was associated with a higher risk of adverse outcomes. This finding may be explained because insulin use may reflect more severe diabetes or longer diabetes duration, and these patients are at higher risk for adverse outcomes in the setting of COVID-19 infection. In our study, because only observational studies were available and some baseline indicators were lacking, partial selection bias could not be ruled out. Patients with severe COVID-19 infection, especially those with respiratory distress, need insulin therapy.^1^ Insulin should always be the preferred medication in any emergency situation and can be used at any stage of COVID-19.^6^

### Limitations

The study still has several limitations. First, although this study included a large number of participants, all of the included studies were observational studies. Therefore, the results of this study should be interpreted as associations. Second, the results of this study may be affected by diabetes comorbidities at baseline, baseline glycemic control, diabetes duration, and diabetes type, which were not assessed in this study. Third, differences in vaccination status, COVID-19 variants, and inpatient protocols may affect outcomes. Fourth, because of the complexity of concomitant diabetes medications, analysis of single drugs was not possible.

## Conclusions

This network meta-analysis found that use of SGLT-2is for diabetes before COVID-19 infection was associated with lower COVID-19–related adverse outcomes. In addition to SGLT-2is, GLP-1RAs and metformin were also associated with relatively low risk of adverse outcomes.

## Notes

Supplement. **eAppendix 1.** PRISMA NMA Checklist of items to include when reporting a systematic review involving a network meta-analysis **eAppendix 2.** Research Data Collection Process **eTable 1.** Example of Search Term Strategy **eTable 2.** Risk of Bias Assessment of the Included Studies According to the Newcastle-Ottawa Scale (NOS) **eTable 3.** Global Inconsistency Tests **eTable 4.** The Evaluation of Local Inconsistency by Node-Split Modeling **eTable 5.** Heterogeneity Tests **eTable 6.** Sensitivity Analysis **eFigure 1.** Heat Map Among Glucose-Lowering Therapies for Mortality (Random-Effects Model) **eFigure 2.** The Evaluation of Publication Bias by Funnel Plots **eFigure 3.** Brooks-Gelman-Rubin Diagnosis Plot **eFigure 4.** Meta-regression Analysis for the Mean Age and Male Proportion Between Studies in the Network Meta-analysis
