# Supplementary material for: Association of Glucose-Lowering Drugs With Outcomes in Patients With Diabetes Before Hospitalization for COVID-19: A Systematic Review and Network Meta-analysis
Source: JAMA Netw Open. 2022 Dec 6;5(12):e2244652. doi: 10.1001/jamanetworkopen.2022.44652 (PMC9856231; doi:10.1001/jamanetworkopen.2022.44652)
Supplement: Supplement. — eAppendix 1. PRISMA NMA Checklist of items to include when reporting a systematic review involving a network meta-analysis eAppendix 2. Research Data Collection Process eTable 1. Example of Search Term Strategy eTable 2. Risk of Bias Assessment of the Included Studies According to the Newcastle-Ottawa Scale (NOS) eTable 3. Global Inconsistency Tests eTable 4. The Evaluation of Local Inconsistency by Node-Split Modeling eTable 5. Heterogeneity Tests eTable 6. Sensitivity Analysis eFigure 1. Heat Map Among Glucose-Lowering Therapies for Mortality (Random-Effects Model) eFigure 2. The Evaluation of Publication Bias by Funnel Plots eFigure 3. Brooks-Gelman-Rubin Diagnosis Plot eFigure 4. Meta-regression Analysis for the Mean Age and Male Proportion Between Studies in the Network Meta-analysis [file jamanetwopen-e2244652-s001.pdf]

Supplementary Online Content

Zhu Z, Zeng Q, Liu Q, Wen J, Chen G. Association of glucose-lowering drugs with outcomes in patients with diabetes before hospitalization for COVID-19: a systematic review and network meta-analysis. *JAMA Netw Open*. 2022;5(12):e2244652. doi:10.1001/jamanetworkopen.2022.44652

eAppendix 1. PRISMA NMA Checklist of Items to Include When Reporting a Systematic Review Involving a Network Meta-Analysis

eAppendix 2. Research Data Collection Process

eTable 1. Example of Search Term Strategy

eTable 2. Risk of Bias Assessment of the Included Studies According to the Newcastle-Ottawa Scale (NOS)

eTable 3. Global Inconsistency Tests

eTable 4. The Evaluation of Local Inconsistency by Node-Split Modeling

eTable 5. Heterogeneity Tests

eTable 6. Sensitivity Analysis

eFigure 1. Heat Map Among Glucose-Lowering Therapies for Mortality (Random-Effects Model)

eFigure 2. The Evaluation of Publication Bias by Funnel Plots

eFigure 3. Brooks-Gelman-Rubin Diagnosis Plot

eFigure 4. Meta-regression Analysis for the Mean Age and Male Proportion Between Studies in the Network Meta-Analysis

This supplementary material has been provided by the authors to give readers additional information about their work.

© 2022 Zhu Z et al. *JAMA Network Open.*

eAppendix 1. PRISMA NMA Checklist of items to include when reporting a systematic review involving a network meta-analysis

Section/Topic	Item #	Checklist Item	Reported on Page #
**TITLE**			
Title	1	Identify the report as a systematic review *incorporating a network meta-analysis (or related form of meta-analysis).*	1
**ABSTRACT**			
Structured summary	2	Provide a structured summary including, as applicable:<br>**Background:** main objectives<br>**Methods:** data sources; study eligibility criteria, participants, and interventions; study appraisal; and *synthesis methods, such as network meta-analysis.*<br>**Results:** number of studies and participants identified; summary estimates with corresponding confidence/credible intervals; *treatment rankings may also be discussed. Authors may choose to summarize pairwise comparisons against a chosen treatment included in their analyses for brevity.*<br>**Discussion/Conclusions:** limitations; conclusions and implications of findings.<br>**Other:** primary source of funding; systematic review registration number with registry name.	2<br>2<br>2<br><br><br>2<br><br><br><br><br>2
**INTRODUCTION**			
Rationale	3	Describe the rationale for the review in the context of what is already known, *including mention of why a network meta-analysis has been conducted.*	3
Objectives	4	Provide an explicit statement of questions being addressed, with reference to participants, interventions, comparisons, outcomes, and study design (PICOS).	3
**METHODS**			
Protocol and registration	5	Indicate whether a review protocol exists and if and where it can be accessed (e.g., Web address); and, if available, provide registration information, including registration number.	None
Eligibility criteria	6	Specify study characteristics (e.g., PICOS, length of follow-up) and report characteristics (e.g., years considered, language, publication status) used as criteria for eligibility, giving rationale. *Clearly describe eligible treatments included in the treatment network, and note whether any have been clustered or merged into the same node (with justification).*	3-4
Information sources	7	Describe all information sources (e.g., databases with dates of coverage, contact with study authors to identify additional studies) in the search and date last searched.	3
Search	8	Present full electronic search strategy for at least one database, including any limits used, such that it could be repeated.	eTable 1
Study selection	9	State the process for selecting studies (i.e., screening, eligibility, included in systematic review, and, if applicable,	Figure 1

© 2022 Zhu Z et al. *JAMA Network Open.*

		included in the meta-analysis).	
Data collection process	10	Describe method of data extraction from reports (e.g., piloted forms, independently, in duplicate) and any processes for obtaining and confirming data from investigators.	3-4 and eAppendix2
Data items	11	List and define all variables for which data were sought (e.g., PICOS, funding sources) and any assumptions and simplifications made.	3
**Geometry of the network**	**S1**	Describe methods used to explore the geometry of the treatment network under study and potential biases related to it. This should include how the evidence base has been graphically summarized for presentation, and what characteristics were compiled and used to describe the evidence base to readers.	4-5
Risk of bias within individual studies	12	Describe methods used for assessing risk of bias of individual studies (including specification of whether this was done at the study or outcome level), and how this information is to be used in any data synthesis.	4
Summary measures	13	State the principal summary measures (e.g., risk ratio, difference in means). *Also describe the use of additional summary measures assessed, such as treatment rankings and surface under the cumulative ranking curve (SUCRA) values, as well as modified approaches used to present summary findings from meta-analyses.*	4-5
Planned methods of analysis	14	Describe the methods of handling data and combining results of studies for each network meta-analysis. This should include, but not be limited to:<br>• *Handling of multi-arm trials;*<br>• *Selection of variance structure;*<br>• *Selection of prior distributions in Bayesian analyses; and*<br>• *Assessment of model fit.*	5
**Assessment of Inconsistency**	**S2**	Describe the statistical methods used to evaluate the agreement of direct and indirect evidence in the treatment network(s) studied. Describe efforts taken to address its presence when found.	5
Risk of bias across studies	15	Specify any assessment of risk of bias that may affect the cumulative evidence (e.g., publication bias, selective reporting within studies).	5
Additional analyses	16	Describe methods of additional analyses if done, indicating which were pre-specified. This may include, but not be limited to, the following:<br>• Sensitivity or subgroup analyses;<br>• Meta-regression analyses;<br>• *Alternative formulations of the treatment network; and*<br>• *Use of alternative prior distributions for Bayesian analyses (if applicable).*	5

© 2022 Zhu Z et al. *JAMA Network Open.*

RESULTS†

Study selection	17	Give numbers of studies screened, assessed for eligibility, and included in the review, with reasons for exclusions at each stage, ideally with a flow diagram.	5, Figure 1
**Presentation of network structure**	**S3**	Provide a network graph of the included studies to enable visualization of the geometry of the treatment network.	5, Figure 2
**Summary of network geometry**	**S4**	Provide a brief overview of characteristics of the treatment network. This may include commentary on the abundance of trials and randomized patients for the different interventions and pairwise comparisons in the network, gaps of evidence in the treatment network, and potential biases reflected by the network structure.	5
Study characteristics	18	For each study, present characteristics for which data were extracted (e.g., study size, PICOS, follow-up period) and provide the citations.	5,Table 1
Risk of bias within studies	19	Present data on risk of bias of each study and, if available, any outcome level assessment.	eTable 2
Results of individual studies	20	For all outcomes considered (benefits or harms), present, for each study: 1) simple summary data for each intervention group, and 2) effect estimates and confidence intervals. *Modified approaches may be needed to deal with information from larger networks.*	eTable 4
Synthesis of results	21	Present results of each meta-analysis done, including confidence/credible intervals. *In larger networks, authors may focus on comparisons versus a particular comparator (e.g. placebo or standard care), with full findings presented in an appendix. League tables and forest plots may be considered to summarize pairwise comparisons.* If additional summary measures were explored (such as treatment rankings), these should also be presented.	6, Figure3 and e Figure 1
**Exploration for inconsistency**	**S5**	Describe results from investigations of inconsistency. This may include such information as measures of model fit to compare consistency and inconsistency models, *P* values from statistical tests, or summary of inconsistency estimates from different parts of the treatment network.	6, eTable 3 and eTable 4
Risk of bias across studies	22	Present results of any assessment of risk of bias across studies for the evidence base being studied.	6, eTable 5
Results of additional analyses	23	Give results of additional analyses, if done (e.g., sensitivity or subgroup analyses, meta-regression analyses, *alternative network geometries studied, alternative choice of prior distributions for Bayesian analyses,* and so forth).	7, eTable 6

DISCUSSION

Summary of evidence	24	Summarize the main findings, including the strength of evidence for each main outcome; consider their relevance to key groups (e.g., healthcare providers, users, and policy-makers).	7-8
Limitations	25	Discuss limitations at study and outcome level (e.g., risk of bias), and at review level (e.g., incomplete retrieval of identified research, reporting bias). *Comment on the validity of the assumptions, such as transitivity and consistency. Comment*	8

© 2022 Zhu Z et al. *JAMA Network Open.*

		on any concerns regarding network geometry (e.g., avoidance of certain comparisons).	
Conclusions	26	Provide a general interpretation of the results in the context of other evidence, and implications for future research.	8
**FUNDING**			
Funding	27	Describe sources of funding for the systematic review and other support (e.g., supply of data); role of funders for the systematic review. This should also include information regarding whether funding has been received from manufacturers of treatments in the network and/or whether some of the authors are content experts with professional conflicts of interest that could affect use of treatments in the network.	9

PICOS = population, intervention, comparators, outcomes, study design.
* Text in italics indicates wording specific to reporting of network meta-analyses that has been added to guidance from the PRISMA statement.
† Authors may wish to plan for use of appendices to present all relevant information in full detail for items in this section.

eAppendix 2. Research data collection process

Item of collection process	Specific measures
**1. Eligibility criteria**	
1.1 Types of studies	All articles reporting on observational studies and randomized controlled trials were included. Reviews, comments, editorials, letters to the editor were excluded. Letters needed to be carefully assessed for available data.
1.2 Publication restrictions	Without language limitation (published in English, Chinese, German, etc). Without any regional and publication restrictions.
1.3 Types of participants	All diabetes hospitalized for COVID-19, either male or female, and across all ages. Pregnant women and those younger than 14 years were excluded. In the setting of the epidemic, the analysis on diabetes type, diabetes severity and diabetes duration was lacking.
1.4 Types of intervention	(At least 2 of the following glucose-lowering therapies) SGLT-2i, GLP1-RA, metformin, DPP-4i, thiazolidinediones, secretagogues, glycosidasei, and insulin. Patients received glucose-lowering therapies at least a window of 14 days prior to hospitalization.
1.5 Types of outcome measures	A composite adverse outcome including the need for ICU admission, invasive and non-invasive mechanical ventilation, or in-hospital death. If all three outcomes were described, we chose the data of in-hospital death.
**2. Search strategy**	
2.1 Databases	PubMed, Embase, Cochrane Central Register, Web of Science, and ClinicalTrials. Gov. Reference lists of included articles, review articles and meta-analyses were also screened. Grey literature without peer review was also carefully considered.
2.2 Retrieval time	From inception to 5 September 2022
2.3 Search query	eTable 1 in the Supplement.
**3. Study quality assessment**	
3.1 Application method	Blinded review by 2 independent reviewers (Z.Z. and Q.Y.Z.) and consultations of disagreement by a third independent reviewer.
3.2 Quality evaluation	The Newcastle Ottawa scale was used (range from 0 to 9). Studies with a score ≥7 were considered to be included.
3.3 Selection flowchart	Figure 1.
**4. Data acquisition**	Data were obtained from the original articles and supplementary materials. If the same population was in different studies, in order to avoid bias, the most complete data was selected. Besides, we obtained raw data by contacting the corresponding author.
**5. Data extraction**	
5.1 Application method	A unified and independent data extraction table was employed. Blinded review by 2 independent reviewers (Z.Z. and Q.Y.Z.) and consultations of disagreement by a third independent reviewer.
5.2 Extract content	Study information(year, author, country), participant characteristics at baseline （age, sex, BMI, HbA1c, diabetes duration), total number and death number of people using each glucose-lowering therapy.
5.3 Retrieval time	From inception to 5 September 2022
5.4 Unified standard	We expressed the data using mean and percentage. When the mean and percentage of baseline characteristics were not provided, the percentage of BMI ≥30kg/m2, HbA1c≥ 7.5%, and diabetes duration≥10 years were calculated.
5.5 Included in NMA	Total number and death number of people using each glucose-lowering therapy were included in the network meta-analysis.

© 2022 Zhu Z et al. *JAMA Network Open.*

eTable 1. Example of search term strategy

MEDLINE	N
1# ((2019 novel coronavirus)or(COVID-19)or(SARS-CoV-2)or(2019-nCoV)or(coronavirus disease 2019)or(coronavirus disease-19)or(COVID19)	292,186
2# ((Diabete)or(Diabetes Mellitus)or(Prediabetic)or(Diabetic Ketoacidosis)or(Donohue Syndrome)or(Ketoacidosis))or((Niddm)or(Mody)or(IDDM)or(Diabetic)or(DIDMOAD)or(Wolfram)or(Leprechaunism)or(Mendenhall)or(Prediabete)))	912,858
3# (Antihyperglycemics)or(Hypoglycemics)or(Antidiabetics)or(Biguanides）or(Butylbiguanide)or(1-Butylbiguanide)or(Adebit)or(Gliporal)or(Silubin)or(Chlorhexidine)or(Tubulicid)or(Phenformin)or(Phenylethylbiguanide)or(Fenformin)or(Proguanil)or(Chloroguanide)or(Paludrine)or(Paludrin)or(Chloriguane)or(Glucophage)or(Dimethylbiguanidine)or(Metformin)or(SGLT 2 Inhibitors)or(Sodium-Glucose Transporter 2 Inhibitor)or(Sodium Glucose Transporter 2 Inhibitors)or(SGLT2 Inhibitors)or(SGLT-2 Inhibitors)or(Gliflozins)or(GLP-1 Receptor)or(Glucagon Like Peptide 1 Receptor)or(GLP-1R)or(GLP 1R)or(GLP1R)or(liraglutide)or(loxenatide)or(semaglutide)or(lixisenatide)or(Dipeptidyl-Peptidase IV Inhibitors)or(DPP-4I)or(DPP4I)or(DPP-4 Inhibitors)or(alogliptin)or(anagliptin)or(diprotin A)or(Linagliptin)or(lipoyl vildagliptin)or(methionyl-2-cyanopyrrolidine)or(saxagliptin)or(Sitagliptin Phosphate)or(sulphostin)or(Vildagliptin)or(Sulfonylurea)or(Dymelor)or(Carbutamide)or(Glybutamide)or(Aminophenurobutane)or(Butylcarbamide)or(Bucarban)or(Oranyl)or(Glucamide)or(Chlorpropamide)or(Glyclazide)or(Diamicron)or(Glyade)or(Glipizide)or(Glypidizine)or(Glidiazinamide)or(Glydiazinamide)or(Glucotrol)or(Minidiab)or(Ozidia)or(Glyburide)or(Glybenclamide)or(Glibenclamide)or(Diabeta)or(Euglucon 5)or(Neogluconin)or(Maninil)or(Daonil)or(Tolazamide)or(Tolbutamide)or(Artosin)or(Diabetol)or(Diaval)or(Orinase)or(Rastinon)or(Thiazolidinediones)or(Pioglitazone)or(Actos) or(Rosiglitazone)or(Avandia)or(Troglitazone)or(Insulin)	577,596
1# and 2# and 3#	1,166

© 2022 Zhu Z et al. *JAMA Network Open.*

eTable 2. Risk of bias assessment of the included studies according to the Newcastle-Ottawa Scale (NOS)

First author	Study design	Selection	Comparability	Outcome	Total score	Result
Elibo	Case-Control	***	**	***	8	Good
Luo	Case-Control	****	**	***	9	Good
Ong	Case-Control	**	**	***	7	Good
Shestakova	Case-Control	**	**	***	7	Good
Zhang Q	Case-Control	**	**	***	7	Good
Nafakhi	Case-Control	**	**	***	7	Good
Mirani	Case-Control	****	**	***	9	Good
Khunti	Cohort	****	**	***	9	Good
Yan	Cohort	***	**	***	8	Good
Israelsen	Cohort	***	**	**	7	Good
Pérez-Belmonte	Cohort	****	**	***	9	Good
Li	Cohort	***	**	***	8	Good
Wargny	Cohort	***	**	***	8	Good
Chen	Cohort	***	**	***	8	Good
Crouse	Cohort	****	**	***	9	Good
Lally	Cohort	***	**	***	8	Good
Zhang JY	Cohort	****	**	***	9	Good
Cariou	Cohort	***	**	***	8	Good
Hayek	Cohort	***	**	***	8	Good
Kahkoska	Cohort	***	**	***	8	Good
Luk	Cohort	***	**	***	8	Good
Sourij	Cohort	***	**	***	8	Good
Nyland	Cohort	**	**	***	7	Good
Orioli	Cohort	***	**	***	8	Good
Pazoki	Cohort	***	**	***	8	Good
Ramos-Rincón	Cohort	***	**	***	8	Good
Yu	Cohort	****	**	***	9	Good
Silverii	Cohort	**	**	***	7	Good
Cheng	Cohort	**	**	***	7	Good
Yeh	Cohort	***	**	***	8	Good
Min	Cohort	**	**	***	7	Good

© 2022 Zhu Z et al. *JAMA Network Open.*

eTable 3. Global inconsistency tests

Consistency tests	Consistent model	Inconsistent model
Dbar	148.3632	148.2561
pD	90.91439	92.36368
DIC	239.27758	240.61978
dDIC	1.3422	

Abbreviations: dDIC: The difference of Deviance Information Criterion (dDIC)
Note: dDIC＜5 represents good global consistency.

eTable 4. The evaluation of local inconsistency by node-split modeling

Comparison	Direct Effect	Indirect Effect	Network Effect	P-value
Metformin.DPP4i	0.28 (-0.021, 0.57)	1.0 (0.16, 1.9)	0.41 (0.15, 0.66)	0.10555
Metformin.GLP1RA	0.020 (-0.42, 0.45)	-0.19 (-0.78, 0.40)	-0.031 (-0.36, 0.30)	0.570425
Metformin.SGLT2i	-0.12 (-0.55, 0.30)	-0.36 (-1.0, 0.29)	-0.20 (-0.54, 0.14)	0.5438
Metformin.Thiazolidinediones	0.20 (-0.37, 0.79)	0.042 (-0.92, 1.0)	0.10 (-0.33, 0.54)	0.7704
DPP4i.Glycosidasei	0.28 (-0.42, 1.0)	0.038 (-0.93, 1.0)	-0.10 (-0.55, 0.36)	0.692
DPP4i.Insulin	0.22 (-0.0017, 0.47)	0.26 (-0.39, 0.92)	0.31 (0.047, 0.58)	0.9158
DPP4i.Secretagogues	-0.16 (-0.45, 0.15)	0.22 (-1.6, 1.9)	-0.23 (-0.51, 0.049)	0.672125
GLP1RA.Glycosidasei	1.2 (0.093, 2.2)	0.091 (-0.47, 0.65)	0.33 (-0.16, 0.83)	0.0815
GLP1RA.Insulin	0.42 (-0.0050, 0.84)	1.3 (0.76, 1.9)	0.74 (0.42, 1.1)	0.013075
GLP1RA.Secretagogues	0.16 (-0.28, 0.61)	0.32 (-0.33, 0.96)	0.20 (-0.14, 0.54)	0.681575
GLP1RA.SGLT2i	-0.17 (-0.57, 0.22)	0.24 (-0.82, 1.3)	-0.17 (-0.54, 0.20)	0.466725
GLP1RA.Thiazolidinediones	0.32 (-0.24, 0.90)	0.065 (-1.0, 1.1)	0.13 (-0.33, 0.59)	0.6539
Glycosidasei.Secretagogues	-0.52 (-1.1, 0.066)	-0.41 (-1.6, 0.76)	-0.13 (-0.59, 0.32)	0.869625
Glycosidasei.SGLT2i	-1.6 (-2.6, -0.59)	-0.094 (-0.70, 0.49)	-0.50 (-1.0, 0.0024)	0.014025
Glycosidasei.Thiazolidinediones	-0.66 (-1.6, 0.29)	-0.012 (-0.80, 0.76)	-0.20 (-0.77, 0.36)	0.2995
Insulin.Secretagogues	-0.52 (-0.82, -0.24)	-0.49 (-1.3, 0.31)	-0.54 (-0.82, -0.27)	0.945975
Insulin.SGLT2i	-0.73 (-1.2, -0.27)	-1.0 (-1.6, -0.45)	-0.91 (-1.3, -0.57)	0.435275
Insulin.Thiazolidinediones	-0.60 (-1.2, 0.014)	-0.53 (-1.3, 0.22)	-0.61 (-1.1, -0.17)	0.8939
Secretagogues.SGLT2i	-0.45 (-0.91, 0.0017)	-0.24 (-0.92, 0.46)	-0.37 (-0.73, -0.019)	0.6058
Secretagogues.Thiazolidinediones	-0.080 (-0.66, 0.50)	-0.094 (-1.1, 0.87)	-0.070 (-0.52, 0.38)	0.983
SGLT2i.Thiazolidinediones	0.28 (-0.34, 0.89)	0.35 (-0.54, 1.2)	0.30 (-0.17, 0.78)	0.89225

eTable 5. Heterogeneity tests

Treatment 1	Treatment 2	I2.Direct	I2.Indirect	P.Inconsistent
Metformin	DPP4i	66.44	43.49	NA
Metformin	GLP1RA	0.00	38.71	0.13
Metformin	Glycosidasei	0.00	0.00	NA
Metformin	Insulin	80.92	76.55	NA
Metformin	Secretagogues	0.00	0.00	NA
Metformin	SGLT2i	85.36	84.82	0.73
Metformin	Thiazolidinediones	0.00	0.00	0.24
DPP4i	GLP1RA	35.65	77.76	NA
DPP4i	Glycosidasei	0.00	0.00	0.44
DPP4i	Insulin	54.79	69.14	0.00
DPP4i	Secretagogues	0.00	0.00	0.06
DPP4i	SGLT2i	35.71	74.71	NA
DPP4i	Thiazolidinediones	0.00	0.00	NA
GLP1RA	Glycosidasei	0.00	0.00	0.58
GLP1RA	Insulin	66.97	60.10	0.12
GLP1RA	Secretagogues	0.00	0.00	0.38
GLP1RA	SGLT2i	0.00	0.00	0.42
GLP1RA	Thiazolidinediones	0.00	0.00	0.59
Glycosidasei	Insulin	53.62	73.67	NA
Glycosidasei	Secretagogues	0.00	0.00	0.44
Glycosidasei	SGLT2i	0.00	0.00	0.49
Glycosidasei	Thiazolidinediones	0.00	0.00	0.96
Insulin	Secretagogues	59.05	47.83	NA
Insulin	SGLT2i	82.67	80.73	1.00
Insulin	Thiazolidinediones	0.00	0.00	0.30
Secretagogues	SGLT2i	32.50	72.45	0.05
Secretagogues	Thiazolidinediones	0.00	0.00	0.34
SGLT2i	Thiazolidinediones	0.00	0.00	0.55
**Global I2**		**47.15**	49.19	

eTable 6. Sensitivity analysis

Sensitivity analysis		Sensitivity 1		Sensitivity 2	
		Consistent model	Inconsistent model	Consistent model	Inconsistent model
Consistency tests	Dbar	136.21962	135.99559	119.00704	119.2832
	pD	83.85043	84.93737	65.73074	66.95511
	DIC	220.07005	220.93296	184.73777	186.23831
SUCRA(%)	Metformin	6(19%)		5(36%)	
	DPP4i	2(76%)		2(83%)	
	GLP1RA	7(15%)		6(21%)	
	Glycosidasei	3(59%)		1(98%)	
	Secretagogues	4(46%)		3(62%)	
	Thiazolidinediones	5(35%)		4(46%)	
	Insulin	1(99%)		/	
	SGLT2i	/		7(4%)	

Note:
Sensitivity 1: the lowest SUCRA value (SGLT-2i) was removed.
Sensitivity 2: the highest SUCRA value (insulin) was removed.

© 2022 Zhu Z et al. *JAMA Network Open.*

eFigure 1. Heat map among glucose-lowering therapies for mortality (random-effects model)

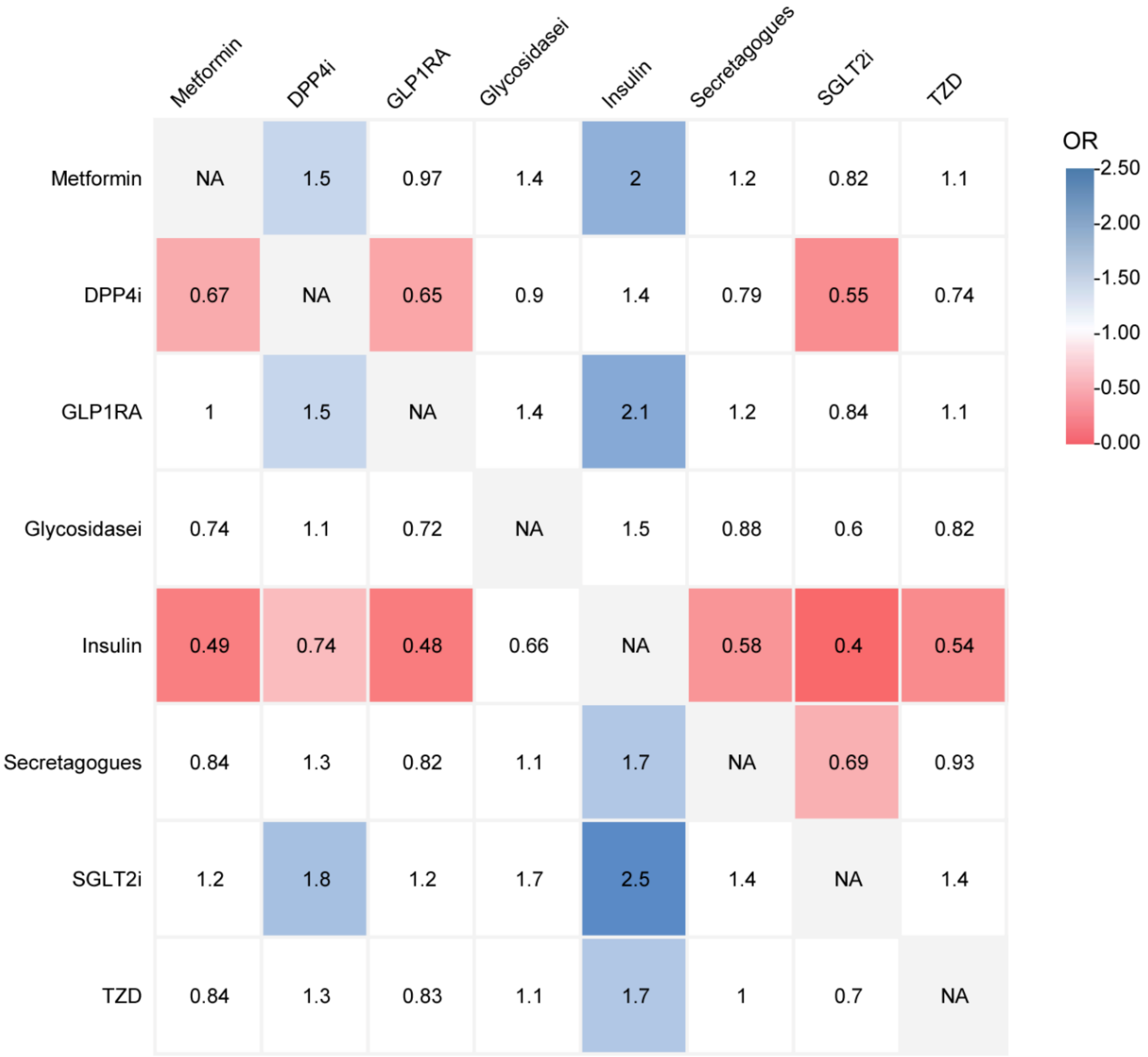

Note: Red indicates the estimates (odds ratio with 95% CI) lower than 1; blue indicates the estimates higher than 1; white indicates the estimates across 1. Grey square indicates that data were not available (NA). Comparisons between treatment must be read from vertical to horizontal and, the estimates lower than 1 indicates fewer adverse events.

© 2022 Zhu Z et al. *JAMA Network Open.*

eFigure 2. The evaluation of publication bias by funnel plots

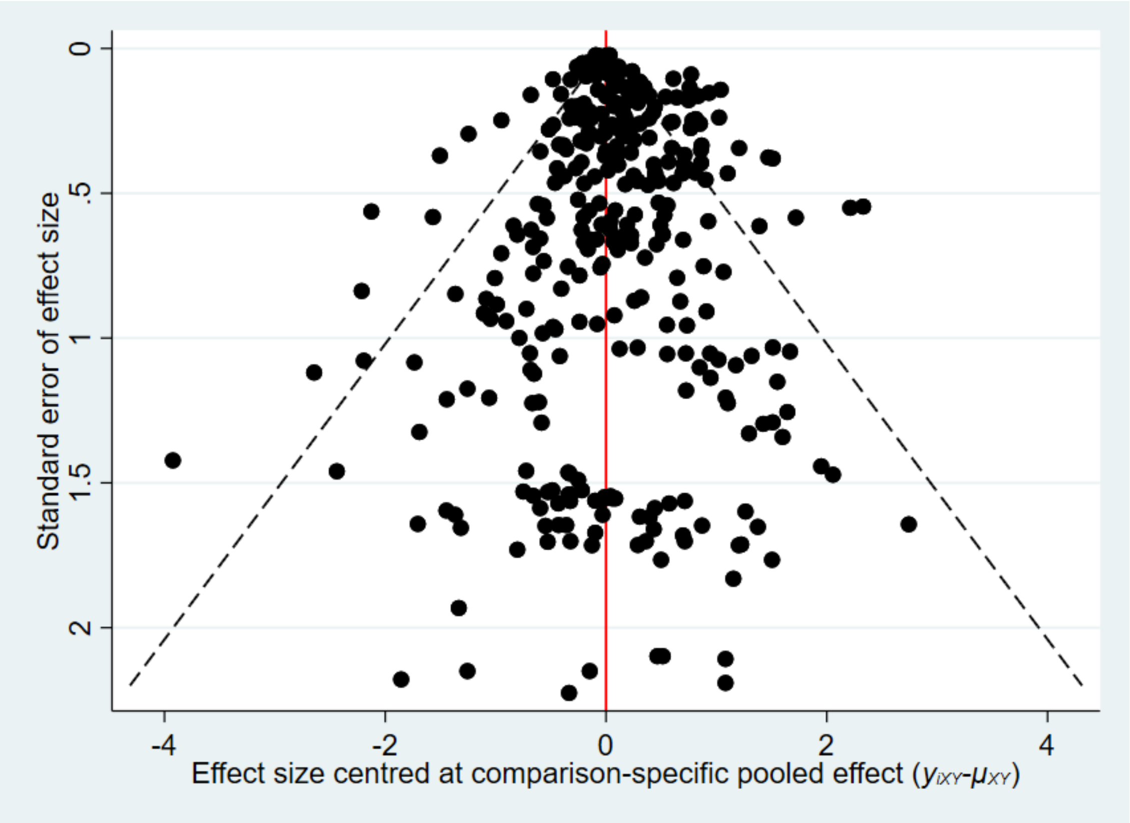

eFigure 3. Brooks-Gelman-Rubin Diagnosis Plot（potential scale reduction factor, PSRF=1）

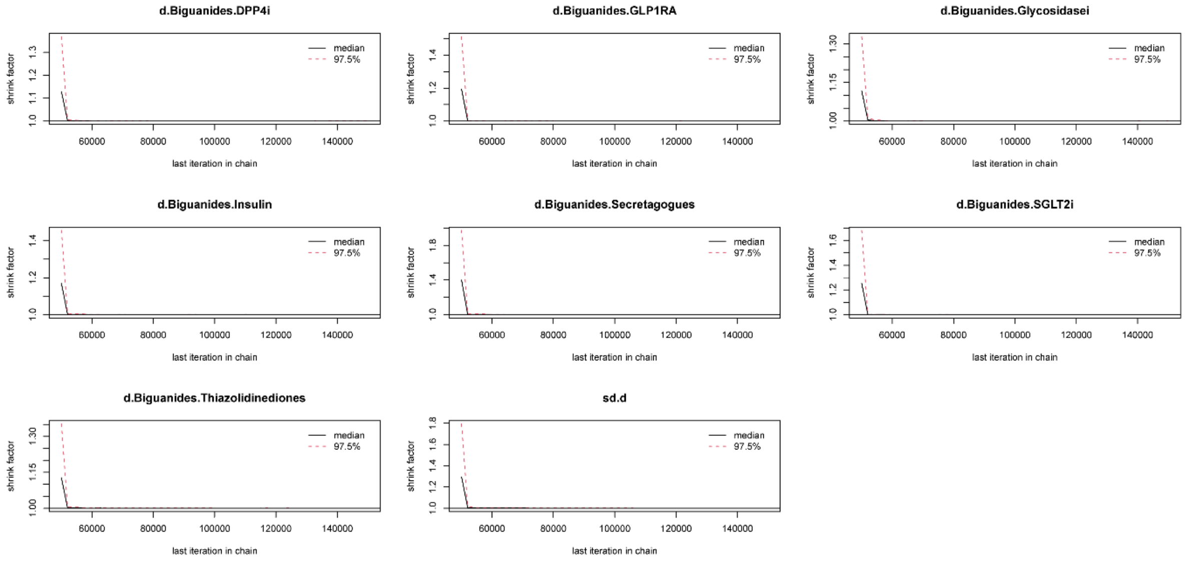

© 2022 Zhu Z et al. *JAMA Network Open.*

eFigure 4. Meta-regression analysis for the mean age and male proportion between studies in the network meta-analysis

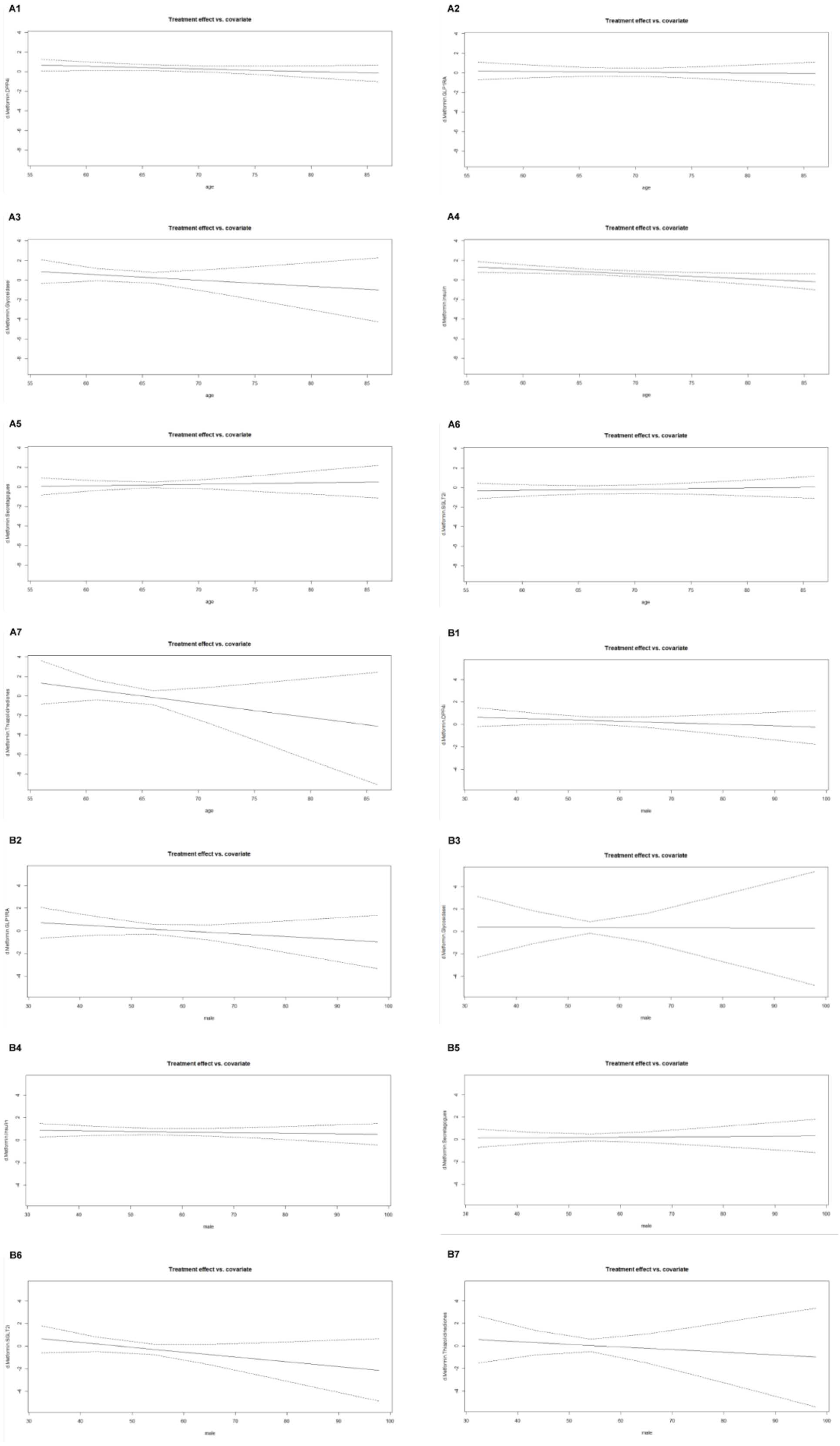

Note: A stands for mean age; (A1) Metformin vs. DPP4i; (A2) Metformin vs. GLP1RA; (A3) Metformin vs. Glycosidasei; (A4) Metformin vs. Insulin; (A5) Metformin vs. Secretagogues; (A6) Metformin vs. SGLT2i; (A7) Metformin vs. Thiazolidinediones.; B stands for male proportion; (B1) Metformin vs. DPP4i; (B2) Metformin vs. GLP1RA; (B3) Metformin vs. Glycosidasei; (B4) Metformin vs. Insulin; (B5) Metformin vs. Secretagogues; (B6) Metformin vs. SGLT2i; (B7) Metformin vs. Thiazolidinediones.

© 2022 Zhu Z et al. *JAMA Network Open.*
